# Paraneoplastic Pemphigus: An Indication for Treatment in Chronic Lymphocytic Leukemia

**DOI:** 10.7759/cureus.8316

**Published:** 2020-05-27

**Authors:** Ifeanyichukwu Onukogu, Preethi Ramachandran, Joshua Narh, Sonu Sahni, Gardith Joseph

**Affiliations:** 1 Internal Medicine, Brookdale University Hospital and Medical Center, New York, USA; 2 Oncology, Brookdale University Hospital and Medical Center, Brooklyn, USA; 3 Internal Medicine, Brookdale University Hospital Medical Center, New York, USA; 4 Research Medicine, New York Institute of Technology College of Osteopathic Medicine, New York, USA; 5 Primary Care, Touro College of Osteopathic Medicine, New York, USA; 6 Oncology, Mount Sinai Medical Center, Brooklyn, USA; 7 Hematology and Oncology, Brookdale University Hospital and Medical Center, Brooklyn, USA

**Keywords:** chronic lymphocytic leukemia, pemphigoid like lesion, paraneoplasm, skin lesions, paraneoplastic syndrome, chemotherapy

## Abstract

Paraneoplastic disorders are rare multiorgan diseases associated with hematological malignancies such as chronic lymphocytic leukemia (CLL). Some of these paraneoplasms manifest as cutaneous lesions, appearing as a simple rash, ulcers or skin thickening. The pathogenesis for this process has been described as development of certain autoimmune reactions against cell wall antigens and proteins. An example is paraneoplastic pemphigus (PNP) which manifests as cutaneous bullae. Bullae may occur anytime during the course of the malignancy i.e. acute phase or remission. Diagnosis involves evaluation of clinical findings, serology and presence of characteristic histological findings. Its pathogenesis is described as development of auto-antibodies against cell junctional and basement membrane proteins. Presence of paraneoplasms has been associated with poorer prognosis and increased mortality in hematological malignancies including CLL. Currently, there are established indications for the treatment of CLL; however, presence of paraneoplasms as an indication for treatment is unclear. Patients with paraneoplasms who underwent expeditious treatment have exhibited better clinical outcomes. Herein we describe a case of a CLL patient in remission presenting with PNP and its response to treatment.

## Introduction

Chronic lymphocytic leukemia (CLL) is a hematological malignancy of clonal B cells characterized by the accumulation of monoclonal B cell lymphocytes in the blood. CLL is the most common of the adult leukemias in the western world [1, 2]. It constitutes about 25 to 30% of all leukemias in the United States with more than 17,000 new cases reported every year, mostly men with estimated median age of 70 [2, 3]. If treated, CLL has a five-year survival rate of over 76% [4]. However, not all CLL is treated immediately unless they meet the criteria for the indications to treat [5].

Paraneoplasms are rare multi-organ diseases associated with neoplasia mainly of lymphoproliferative origin such as leukemia, lymphoma, Castleman’s disease, etc. They are characterized by autoantibodies-antigen mediated manifestations. These paraneoplasms may present as cutaneous lesions, commonly referred to as cutaneous variant paraneoplasms. Examples include bullous pemphigoid, erythema multiforme, graft versus host disease, lichen planus and eosinophilic dermatoses of hematological malignancy among others [6]. The diagnosis of these paraneoplasms depends on the correlation between clinical manifestations and histopathological findings. Paraneoplastic pemphigus (PNP) is a cutaneous variant of CLL. Its pathogenesis is similar to that of regular cutaneous pemphigoids which involves formation of autoantibodies against proteins such as desmoglein-1, desmoglein-3, desmocollin desmoplakin-1, desmoplakin II which are responsible for formation of cell junctions [2, 6].

Diagnosis is primarily made through skin biopsy and direct immunofluorescence showing deposition of IgG at the epithelial basement membrane. In these patients, immunohistochemical stain demonstrates expression of CD-20 and CD-23 which are seen in CLL [7]. Many case reports have shown that patients with PNP have poor prognosis and a high mortality rate reported to be upwards of 75-90% with an average survival of less than one year [5]. Steroids are the recommended first line treatment with a few reports showing response to immunosuppressants such as cyclophosphamide, mycophenolate mofetil, azathioprine, IgG and chemotherapy such as ibrutinib [2, 6, 7]. Herein we present a case of a 70-year-old female with history of CLL in which presentation significant of PNP was indicative of recurrence of disease.

## Case presentation

We present a 79-year-old female with a long-standing history of CLL diagnosed about 10 years ago, who had been on the watch and wait protocol as she was clinically in remission. Collaterally she initially presented to the emergency department (ED) in July of 2014 as a stroke notification. At that time, she was found to have an acute bilateral ischemic cerebrovascular accident with hyperleukocytosis of 200 x 10^9^/L, anemia and thrombocytopenia. At that time of hospitalization, she was also found to have acute myocardial infarction and deep venous thrombosis of the left popliteal vein for which she was treated with dual anti-platelet therapy and full dose anticoagulation. Due to altered mental status in setting of multiple acute pathologies she was intubated for airway protection and admitted to the medical intensive care unit (MICU) for further management. She underwent a total of four weekly cycles of hydroxyurea and rituximab which she tolerated well. Subsequently, she was placed on hydroxyurea. After initial rituximab, she had further treatments for maintenance. By December 2016, it was determined that she was in remission and thus was discontinued on all CLL treatments and recommended to continue clinic follow-up every three to six months. WBC after remission was noted to be less than 10 x 10^9^/L. Home medications consisted of enalapril, sitagliptin, metformin, insulin glargine, atorvastatin and folic acid.

In October 2018, two years after initial ED visit, she presented with a chief complaint of itchy rash on her upper and lower extremities. Physical exam revealed wheelchair bound elderly female, with normal cardiopulmonary exam with skin exam revealing multiple blisters and small bullae on face, neck and forearms with surrounding erythema and desquamation. Laboratory findings are shown below in Table 1. Peripheral flow cytometry findings were consistent with a CD5+ B-cell lymphoproliferative disorder (66% of total cells).

**Table 1:** Laboratory findings during the course of disease progression. ALT: Alanine aminotransferase; ANA: Antinuclear antibody; AST: Aspartate aminotransferase; BP: Bullous pemphigoid; CMV: Cytomegalovirus; EBV: Epstein-Barr virus; HBG: Hemoglobin; HepBsAg: Hepatitis B surface antigen; HCV: Hepatitis C virus; HIV: Human immunodeficiency virus; RBC: Red blood cell count; T-Bil: Total Bilirubin; WCC: White cell count; VZV: Varicella zoster virus.

	Cell Count		Biochemistry	CMP		Viral and Autoimmune Panel	
	Prior to treatment with 1^st^ cycle of Rituximab	At CLL remission following Rituximab	Detection of PNP lesions				
WCC x10^9^/L	200	8.70	26.5	Na	144 mmol/L	HIV	Nonreactive
Neutrophils %	20	41.8	30	K	4.9 mmol/L	EBV titre	0.9
Lymphocytes %	75	50.6	68	Cl	99 mmol/L	CMV antigenemia assay	Negative
Monocytes %	4.5	6.6	2.0	Total Protein	6.4 g/dl	HSV	Negative
Eosinophils %	0.4	0.7	0.0	Albumin	4.4 g/dl	VZV	Negative
Basophils %	0.1	0.3	0.0	T-Bil	0.2 mg/dl	HepBsAg	Negative
RBC x10^12^/L	4.0	3.45	4.27	ALT	11 U/L	HCV	Negative
HBG g/dl	10	12.1	12.9	AST	16 U/L	ANA	1:40
Platelets x10^9^/L	99	155	166	LDH	183 U/L	Kappa free light chains	1.60 mg/dl
				Beta-2-Microglobulin	2.1 mg/L	Lambda free light chains	1.27 mg/dl
						BP 180	>100 U
						BP 230	<5 U

**Figure 1:**
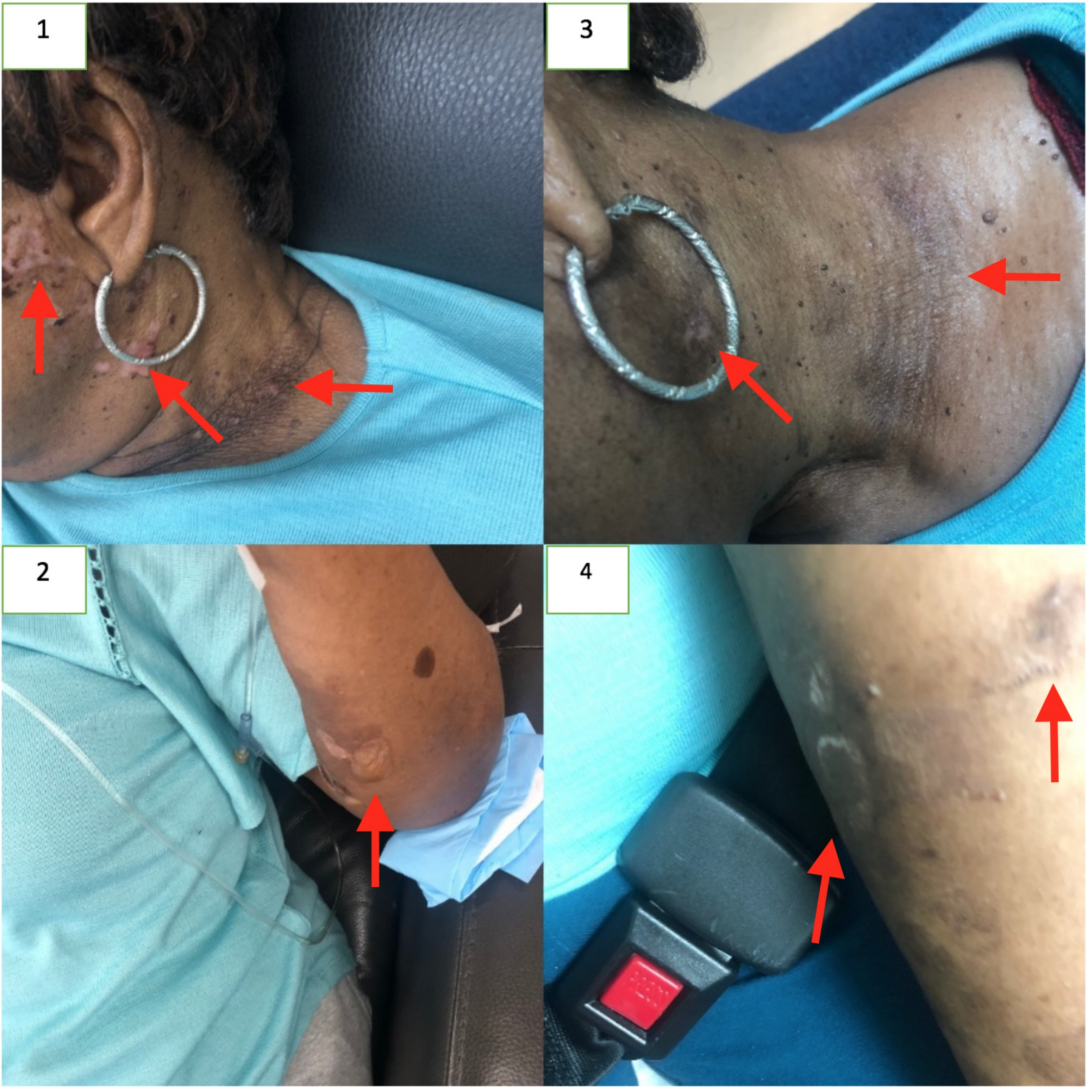
The pemphigus lesions of the neck and forearm before (1 & 2) and after (3 & 4) treatment. Arrows point to lesions of interest.

Skin biopsy was subsequently performed with direct immunofluorescence which noted presence of C3 and IgG aligning the roof and floor of induced blisters. Findings were found to be compatible with pemphigoid family of diseases including bullous pemphigoid and drug-induced pemphigoid. The patient was initially treated with 1 mg/kg of prednisone with transient resolution of cutaneous lesions for a week. Due to the lack of response to steroid therapy, we initiated treatment with the Bruton Tyrosine kinase inhibitor ibrutinib and anti-CD 20+ monoclonal antibody rituximab. The patient is currently in treatment phase with good clinical response which has been shown in Figure 1. The patient continues to follow up in Hematology/Oncology clinic and with Dermatology.

## Discussion

Paraneoplastic pemphigus is a rare autoimmune manifestation of hematological malignancies most commonly lymphoproliferative disorders with some reports indicating an incidence of up to 25% [8]. Although underreported or under diagnosed, identified cases occurred between fifth and eighth decades of life [1]. Paraneoplastic pemphigus is a life-threatening mucocutaneous polymorphic subtype of pemphigus with a variable clinical presentation. Other common etiologies include chemotherapy with drugs such as ibrutinib, purine analogs, and alkylating agents. A few cases have been associated with insect bites through proliferation of eosinophilia by activation of IL4 and IL5 activities [7]. The clinicopathological picture mimics that of pemphigus vulgaris, pemphigus foliaceus, pemphigoid, erythema multiforme, graft versus host disease or lichen planus [9, 10].

The clinical onset of PNP in relation to the underlying hematological malignancy may occur over three timelines. In about two-thirds of cases PNP precedes the detection of the malignancy, with some cases diagnosed during active disease or during remission [11]. A myriad of cases postulate underlying pathogenesis of PNP since its initial discovery. The most widely accepted theory postulates that the malignant process induces both humoral and cell-mediated immunity. The driver mechanism involves the activation of autoantibody production in response to maladaptive immune function and cross reactivity between antibodies directed against tumor cells and epithelial antigens [12]. Autoantibodies against desmoplakins, specifically envoplakin and periplakin, underlie the major antigenic proteins in PNP. These proteins act as epidermal cell adhesion molecules and anchoring proteins for intermediate filaments to desmosomes [13-15]. Less common antigenic proteins include desmoglein-3, desmoplakin-1, desmoplakin II, bullous pemphigoid antigen-1, plectin and protease inhibitor alpha-2-macroglobulin-like-1 (A2ML1). The presence of AntiCD20+ and AntiCD23+ has been detected in PNP due to CLL. The cell-mediated immunity involvement is supported by the detection of CD8+ T lymphocytes, CD56+ and monocytes/macrophages in intraepithelial cell spaces and the dermo-epidermal junction in biopsy samples of affected patients [16]. Other components of the cellular inflammatory process include the production of proinflammatory cytokines such as IL-6, interferon gamma and TNF-alpha which elicit antibody production and CD8+ T lymphocyte activation [17]. The processes described above promote keratinocyte acantholysis resulting in intraepidermal and dermo-epidermal blistering.

Clinical presentation is characterized by the presence of polymorphous lesions evidenced by mucocutaneous papules, pustules, lichenoid plaques, flaccid/tense bullae, desquamative and psoriasiform lesions [11]. Diagnostic modalities for the detection of PNP include lesion skin biopsy for light microscopy and direct immunofluorescence microscopy, serum for ELISA and indirect immunofluorescence. Supportive laboratory tests to diagnose CLL such as complete blood count /with differential, peripheral blood smear, bone marrow aspirate, immunophenotyping and genetic analysis are required as well. Due to the variable histopathological presentation of PNP, a high degree of clinical suspicion with supportive immunohistological findings is required to arrive at a diagnosis.

There is a paucity of data on the treatment of PNP in patients with CLL. Therapeutic modalities that have yielded clinical response include the use of oral glucocorticoids, plasmapheresis, chemotherapy, intravenous immunoglobulins, and myeloablation with anti-CD52+ monoclonal antibody alemtuzumab [18, 19]. Recent evidence has shown clinical response to the combination of Bruton Tyrosine kinase inhibitor Ibrutinib and anti-CD 20+ monoclonal antibody rituximab in patients with CD5+ B-cell chronic lymphocytic leukemia and PNP [20].

## Conclusions

Herein we presented a case of a 79-year-old female with history of CLL who presented with PNP, representing a paraneoplasm. In this case, the skin lesions were identified as PNP and was an indicator of disease recurrence. As demonstrated cutaneous lesions are a possible manifestation of paraneoplasms in the setting of CLL. These lesions can occur secondary to hematological disease, chemotherapy side effects or even insect bites. Thus, diagnostic differentiation among these lesions is a challenge but is necessary to guide therapy. Indirect immunofluorescence has been shown to be most specific for diagnostic accuracy. The proposed pathogenesis involves activation of both humoral and cell-mediated immunities leading to proliferation of auto antibodies against tumor cell surface and epithelial antigens. Most cases of PNP have demonstrated good clinical response to steroids, chemotherapy, immunoglobulins and immunosuppressant depending on the severity and stage of disease. Clinicians should be aware of PNP and its clinical significance in the setting of CLL.
